# Bay Leaf Extract-Based Near-Infrared Fluorescent Probe for Tissue and Cellular Imaging

**DOI:** 10.3390/jimaging7120256

**Published:** 2021-11-30

**Authors:** Benilde Adriano, Nycol M. Cotto, Neeraj Chauhan, Vinita Karumuru, Meena Jaggi, Subhash C. Chauhan, Murali M. Yallapu

**Affiliations:** 1 Department of Immunology and Microbiology, School of Medicine, University of Texas Rio Grande Valley, McAllen, TX 78504, USA; benilde.adriano01@utrgv.edu (B.A.); nycol.cotto01@utrgv.edu (N.M.C.); neeraj.chauhan@utrgv.edu (N.C.); vkarumuru07@gmail.com (V.K.); meena.jaggi@utrgv.edu (M.J.); subhash.chauhan@utrgv.edu (S.C.C.); 2 South Texas Center of Excellence in Cancer Research, School of Medicine, University of Texas Rio Grande Valley, McAllen, TX 78504, USA

**Keywords:** near-infrared imaging, chlorophyll, dietary leaf, cancer, nanoparticles

## Abstract

The development of fluorescence dyes for near-infrared (NIR) fluorescence imaging has been a significant interest for deep tissue imaging. Among many imaging fluoroprobes, indocyanine green (ICG) and its analogues have been used in oncology and other medical applications. However, these imaging agents still experience poor imaging capabilities due to low tumor targetability, photostability, and sensitivity in the biological milieu. Thus, developing a biocompatible NIR imaging dye from natural resources holds the potential of facilitating cancer cell/tissue imaging. Chlorophyll (Chl) has been demonstrated to be a potential candidate for imaging purposes due to its natural NIR absorption qualities and its wide availability in plants and green vegetables. Therefore, it was our aim to assess the fluorescence characteristics of twelve dietary leaves as well as the fluorescence of their Chl extractions. Bay leaf extract, a high-fluorescence agent that showed the highest levels of fluorescence, was further evaluated for its tissue contrast and cellular imaging properties. Overall, this study confirms bay-leaf-associated dye as a NIR fluorescence imaging agent that may have important implications for cellular imaging and image-guided cancer surgery.

## 1. Introduction

Cancer is one of the major leading causes of death in the United States. It has been estimated that in 2021, a total of 1.9 million new cases of cancer and 608,570 deaths will occur in the United States alone [1]. Impactful cancer management relies on early cancer detection but, despite having the readily available equipment in clinical settings for the detection and diagnosis of cancer, such as ultrasounds, X-rays, and magnetic resonance imaging (MRI), the 2021 statistics indicate only a slight increase from those reported in 2010 [2]. This limited improvement may be attributed to the lack of sensitive and specific detection by these methods [3]. 

However, a newer approach known as optical imaging has gained attention as a promising technique for the detection and diagnosis of cancer [4]. Fluorescence imaging is one of the most widely used optical bioimaging methods due to the variety of signal readouts, such as fluorescence intensity, lifetime, quenching, or Förster resonance energy transfer. Conventional fluorescent dyes (cyanine, coumarins, and rhodamines) (i) tend to aggregate, resulting in decreased fluorescence intensity; (ii) face limitation of deep-tissue imaging due to scattered light background; and (iii) produce auto-fluorescence and photo-bleaching [5]. On the other hand, near-infrared (NIR) fluorescence-based imaging, a type of optical imaging, is a noteworthy and safer strategy for cancer cell/tissue imaging compared to radiological imaging. NIR fluorescence holds an advantage over radiological imaging for its assistance in obtaining a better resolution of detection [6,7]. It offers deep tissue penetration and has minimal obstruction by autofluorescence and photon scattering [7]. As a model of optical imaging, NIR is capable of providing information related to the biochemistry and anatomy of any cancerous tissues [4]. There are several NIR dyes, including ICG [8] and IR-1061 [9], that allow high-resolution tissue imaging. Nevertheless, these dyes possess low-quality characteristics that limit their use, namely photo-instability, toxicity, poor water solubility, and short half-lives. On the other hand, NIR fluorescence-emitting materials such as Ag_2_S, and Si nanoparticles, are too large, limiting their use for in vivo applications [10,11]. Therefore, more efficient, effective, and biocompatible alternatives with small particle sizes are urgently required to provide the desired clinical outcomes that can facilitate cancer cell/tissue imaging for improved detection and diagnosis. 

**Scheme 1:**
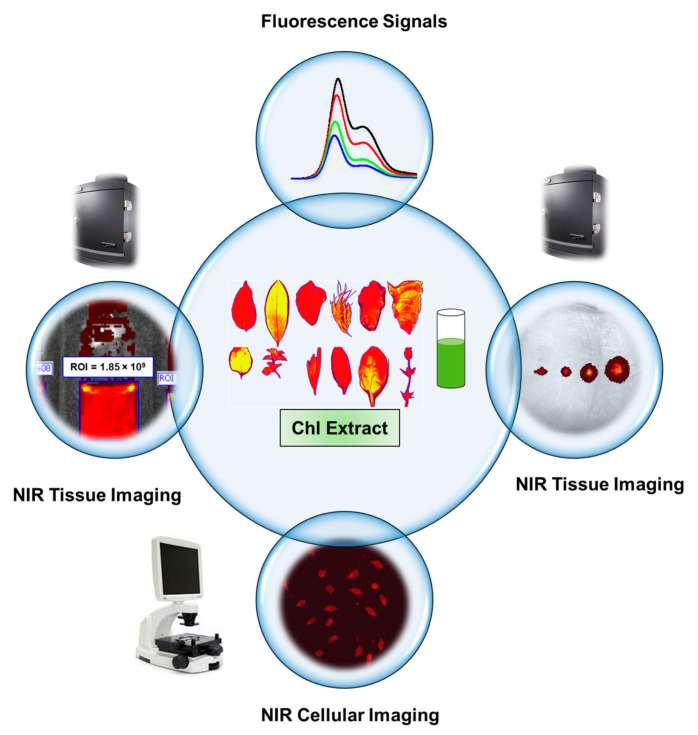
Natural dietary leaf-based NIR fluorescent probe development utilizing leaf chlorophyll extracts and evaluation of their fluorescence properties and proof-of-concept of cellular and tissue imaging capabilities.

Chlorophyll (Chl) is a natural dietary NIR fluorescence-emitting (675 nm) substance that is widely present in plants and green vegetables [12]. It has been observed that plant extracts are rich in Chl and often possess anti-cancer properties, along with other dietary benefits [13,14]. Chlorophyll’s availability, along with its promising assistance for the detection of cancer, makes Chl a potential lead NIR imaging candidate for cancer administration. The in situ synthesis of a NIR carbon dot-based fluorescence probe based on spinach extract using a solvothermal method has been reported [15]. Another study investigated a spinach leaf chlorophyll-based NIR fluorescence composite with octylamine-modified polyacrylic acid. Considering the feasibility of chlorophyll extracts as NIR fluorescent agents, we aim to extract chlorophyll dye from various dietary resources and evaluate its tissue and cancer cell imaging potential (Scheme 1). 

## 2. Materials and Methods

### 2.1. Chemicals and Cell Culture

Twelve different leaves (basil, bay leaf, collard, dill, kale, lettuce, mint, oregano, rosemary, sage, spinach, and thyme) were purchased from Walmart Inc. (Bentonville, AR, USA). All other chemicals and reagents used in this work were purchased from Sigma Aldrich Corporation (St. Louis, MO, USA) unless otherwise mentioned. SK-HEP-1 (Liver cancer) and AsPC-1 (Pancreatic cancer) cell lines were purchased from the American Type Culture Collection (ATCC) (Manassas, VA, USA). These cancer cell lines were maintained in EMEM and RPMI (Gibco, Gibco laboratories, Gaithersburg, MD, USA), respectively, with the following supplements: 10% heat-inactivated FBS (Atlantic Biologicals, Lawrenceville, GA, USA) and 5 mL 1X antibiotic/antimycotic (Sigma, St. Louis, MO, USA). Cell lines were cultured at 37 °C under a humidified atmosphere of 5% CO_2_.

### 2.2. Leaf Extraction Procedure

Each dietary leaf was dried and weighed for 1 g. The leaves were cut into small pieces and placed into individual 50 mL tubes. Next, 6 mL of 70% EtOH was added into each tube and sonicated for 30 s using the VirSonic Ultrasonic Cell Disrupter 100 (SP Industries Company, Warminster, PA, USA). After sonication, the tubes were placed into 1000 mL beakers (to avoid spill) and covered with aluminum foil to avoid contact with light. The beakers holding the 12 samples were then incubated in the Excella E24 Incubator Shaker (New Brunswick Scientific) at 37 °C overnight. The following day, the liquid was extracted from each tube by centrifugation at 2000 rcf for 10 m at 4 °C. After centrifugation, the supernatant of each sample was transferred to new tubes and stored at 4 °C until further use. 

### 2.3. Fluorescence Imaging of Leaf Extracts

An IVIS Imaging System (Perkin Elmer, Waltham, MA, USA) was used to detect fluorescence attributed due to Chl in leaf and leaf extracts with excitation and emission wavelengths at 600 nm and 710 nm, respectively. For the fresh leaf imaging, the leaves were cut into even circles and measured for fluorescence. Fluorescence imaging of leaf extracts was utilized for their fluorescence properties. Each extraction had a stock (control, 1 mg extract/mL), followed by 3 dilutions representing 0.5, 0.25, and 0.166 mg extract/mL concentrations, respectively. A 5 μL drop of these extracts was placed on a filter paper and was imaged in triplicate using similar settings as whole leaf. Acquired images were analyzed using LivingImage 4.5 software provided with the IVIS Imaging System. Data were reported as the radiance (photon emission per unit area) of a region of interest (ROI).

### 2.4. Spectral Analysis

The biochemical characterization of bay leaf extracts was performed with a UV–Vis spectrophotometer at room temperature. The absorption and emission measurements of leaf extract solutions (100 µL of 0.166–1.0 mg/mL solutions) were carried out through placing them in a 96-well plate and analyzing them using a Varioskan LUX multimode microplate reader (Thermo Fisher Scientific, Waltham, MA, USA). The spectral measurement of sample data was collected for absorption (400–800 nm) and fluorescence (excitation λex, 405 nm and emission, λem, 600–850 nm) and analyzed using Skanlt Software RE 6.02.

### 2.5. Agarose Phantom Gel Imaging

To mimic the tissue environment, a 2.5% agarose solution was used to examine the fluorescence of bay leaf extract by dissolving/heating 2.5 g agarose in 100 mL PBS in a microwave over 2–3 min in intervals of 15–25 s. Once completely dissolved, the temperature of agarose was maintained at 80–90 °C on a stirring heat plate to avoid solidification of agarose gels. A stock phantom solution was prepared by mixing 12 μL of the extract in 1200 μL of agarose solution. From this stock solution (1 mg extract/mL), three different dilutions were further prepared (1/2, 1/4, and 1/6). The concentration of agarose solutions was 1, 0.5, 0.25, and 0.166 mg extract/mL, respectively. All samples were placed in clear 2 mL glass vials and allowed to solidify/cool at room temperature. Vials were then imaged for Chl fluorescence using the IVIS Imaging System with excitation and emission at 600/710 nm.

### 2.6. Ex Vivo NIR Imaging

Fresh chicken breast (Walmart Inc. Bentonville, AR, USA) tissue samples were utilized to examine the fluorescence detection of bay leaf extract. The different concentrations of bay leaf extracts of (0.166–1.0 mg/mL) were deposited on chicken tissue and fluorescence images and fluorescence counts were acquired using the IVIS Imaging System and with its associated software. 

### 2.7. Cellular Uptake

SK-HEP-1 (Liver cancer) and AsPC-1 (Pancreatic cancer) cell lines were used to assess the cellular uptake of the bay leaf extract. A six-well plate was used to seed 300,000 cells per well and allowed to attach overnight at 37 °C under a humidified atmosphere of 5% CO_2_. Next day, cells were treated for 1 h at different concentrations (0, 10, 20, 30, 40, and 50 µg) of bay leaf extract. After 1 h, cells were washed with PBS, and the treatment containing medium was replaced with fresh phenol red-free medium before the cells were imaged with a fluorescent microscope (EVOS M7000 Imaging System, ThermoFisher Scientific, Waltham, MA, USA) under the red-light channel. 

### 2.8. Physicochemical Characterization

The Chl extractions were characterized for particle size, total concentration, and surface charge using a Dynamic Light Scattering (DLS) system (Zetasizer Ultra, Malvern Panalytical, Malvern, UK). The samples for particle size and total concentration were prepared using 150 μL of the sample in 3 mL Milli-Q water. The same procedure was repeated for the surface charge, except sample preparation was done with PBS. The samples were sonicated in a plastic tube for 50 s using a VirSonic Ultrasonic Cell Disrupter 100 (SP Industries Company, Warminster, PA, USA) and then transferred to a clear plastic cuvette for concentration and size, or a capillary cuvette for surface charge. All measurements were performed at 25 °C and the results represent the mean of 3 test runs.

### 2.9. Statistical Analysis

All statistical calculations were performed using Origin 6.1 (OriginLab Corp., Northampton, MA, USA). The data are expressed as mean ± standard error of mean (SEM).

## 3. Results

### 3.1. NIR Fluorescence Measurement in Dietary Leaves

**Figure 1:**
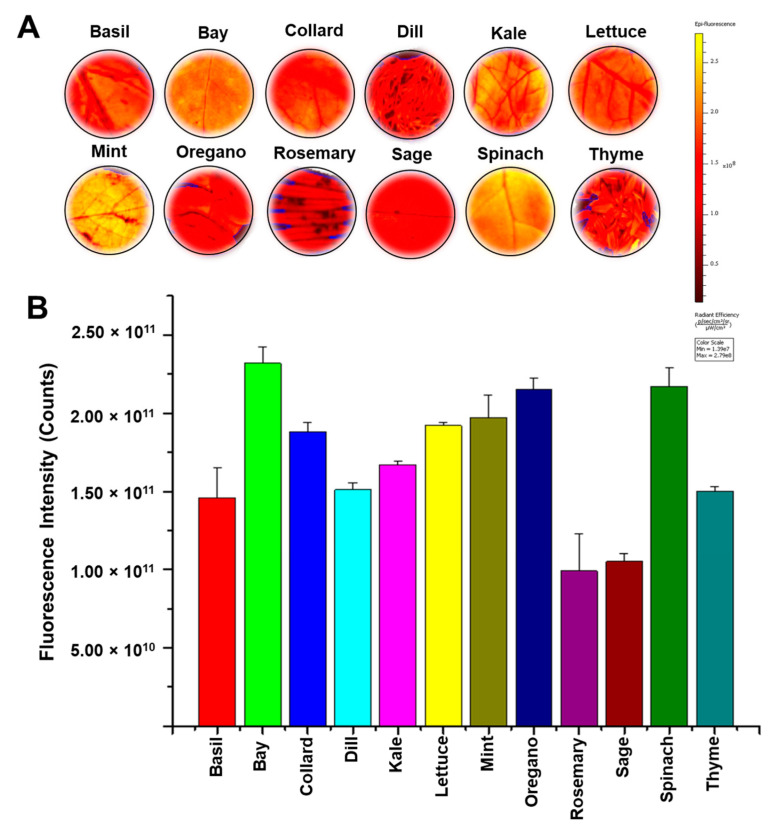
Chlorophyll measurement with IVIS Imaging System. (**A**) Twelve different dietary leaves were imaged using the IVIS Imaging System for chlorophyll content. The chlorophyll fluorescence was measured at an excitation and emission of 600/710 nm. Experiment was performed in triplicate. (**B**) Quantitation of the fluorescence measurement. The figure represents the average of three individual experiments. n = 3.

Initially, we began screening the NIR fluorescence properties of the twelve different dietary leaves using an IVIS Imaging System. Representative images of dietary leaves were imaged in their original form for chlorophyll fluorescence (Figure 1A). The color scale was set to maroon to yellow (low to high fluorescence counts) in the image analysis. The visual observation suggested that bay leaf, collard, lettuce, mint, oregano, and spinach leaves exhibited higher fluorescence compared to the other group of leaves (basil, dill, kale, rosemary, sage, and thyme). The fluorescence signal intensity was measured using software on the leaves (ROI) in photons per second per square centimeter per steradian (p/s·cm^2^·sr) (Figure 1B).

Additionally, a similar fluorescence characteristic of leaves (whole leaves) was observed (Supplementary Materials, Figure S1). This further supports the notion that the quantified fluorescence intensities were higher for the six leaves mentioned above. The confirmed fluorescence order is bay leaf > spinach > oregano > lettuce > collard. To develop a NIR imaging agent with high fluorescence capacity, we chose high NIR property exhibiting leaves (bay leaf, collard, lettuce, mint, oregano, and spinach).

### 3.2. Bay Leaf Extract Exhibits Superior NIR Fluorescence Properties 

**Figure 2:**
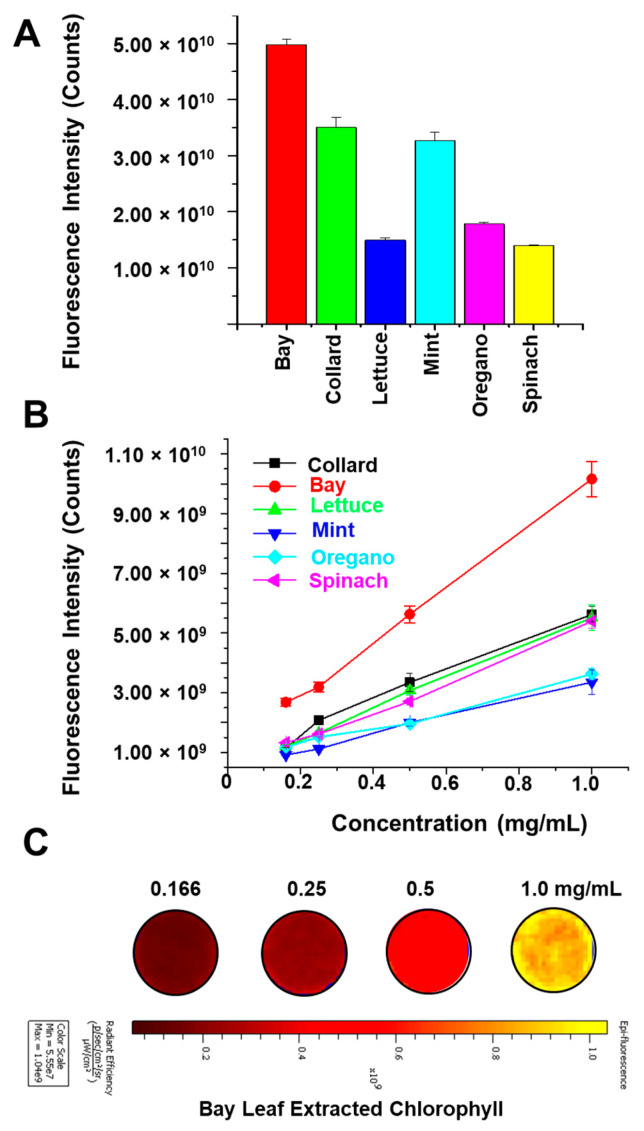
Fluorescence measurement of top six leaf extracts. (**A**) Based upon whole leaf imaging data, six most fluorescent leaves were selected for further imaging experiments. Bar graph represents chlorophyll fluorescent intensity. Bay leaf exhibited the highest fluorescence levels. (**B**) Chlorophyll dye was extracted using EtOH from these leaves. Different dilutions of extracted chlorophyll were prepared and a 5 µL drop was imaged. Line graph represents the average fluorescent intensity of extracted chlorophyll dye at different dilutions from three individual experiments. n = 3. (**C**) Images represent bay leaf extracted chlorophyll concentrations.

To identify a superior fluorescence property from the origins of Chl, the leaf extract was processed for fluorescence measurements. These whole leaf extracts (5 µL drop) were placed on Whatman filter paper and imaged for fluorescence. The physical appearance of NIR fluorescence and their respective counts are presented in Figure 2A,B. Results indicated that bay leaf showed the highest fluorescence intensity. This phenomenon was true at all concentrations (0.166 to 1.0 mg extract/mL) (Figure 2B,C). There was obvious evidence that bay leaf exhibited the highest fluorescence intensity, and the order was bay leaf > collard > mint >oregano > lettuce > spinach. It is important to note that spinach as a whole leaf provided significant fluorescence but not as a leaf extract (Figure 2A). This may be due to the lower amount of Chl during the extraction process. However, it is consistent that bay leaf produced higher NIR fluorescence characteristics.

### 3.3. Bay Leaf Extract Demonstrates Optical Characteristics

**Figure 3:**
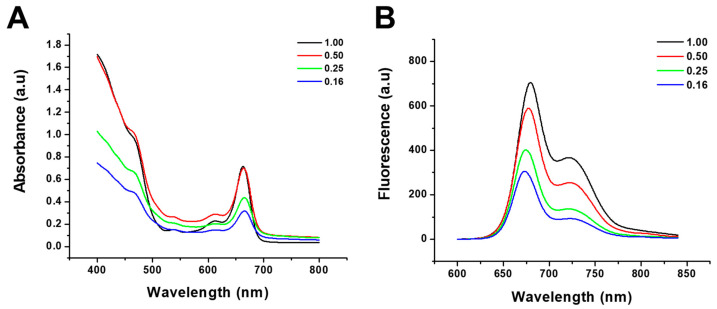
Optical properties of bay leaf extracts (concentration: 0.16–1.0 mg/mL). (**A**) Visible absorption spectra of bay leaf (400–800 nm) and (**B**) fluorescence spectra of bay leaf, excitation: λex, 405 nm and emission: λem, 600–850 nm. Data are representative of a triplicate reading.

The optical properties of bay leaf extracts were determined by visible absorption and fluorescence emission. As shown in Figure 3A, the absorption spectra of bay leaf extracts displayed a blue absorption maximum of ~482 nm and red absorption maxima of ~612 and 661 nm. It is evident from previous studies that a 660 nm absorption peak is a characteristic peak of chlorophyll of various leaf extracts. The range of absorption intensity was in proportion to the concentration of extract. The fluorescence spectra exhibited bay leaf extract emission peaks at 674–680 nm and 725 nm (Figure 3B). These peaks arose due to protochlorophyllide and chlorophyllide in leaf extracts. Thus, these data confirm that the bay leaf extracts possessed a chlorophyll-rich composition. 

### 3.4. Bay Leaf Extract Demonstrates Optical Characteristics

**Figure 4:**
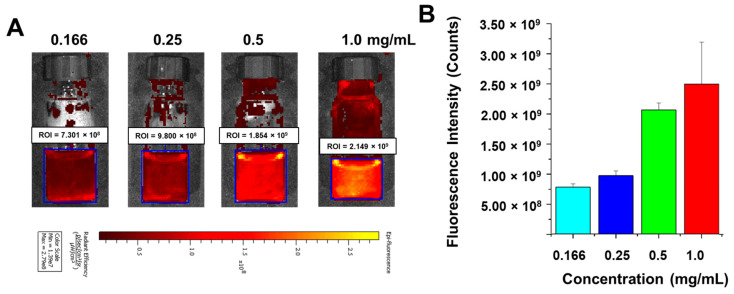
Chlorophyll measurement in agarose gels. Here, 2.5% agarose was utilized for phantom gel preparation with different dilutions of bay leaf extracted chlorophyll. (**A**) Gels were imaged using IVIS Imaging System at 600/710 nm. (**B**) Quantitation of the fluorescence measurement. Data represent the average of three different experiments.

To test the fluorescence property of bay leaf Chl extraction in a tissue-like environment, a 2.5% agarose solution (agar phantom) was used. The agar phantoms were prepared with dilutions of bay leaf extracted Chl. Each dilution was imaged under the excitation and emission of 600/710 nm with the IVIS Imaging System. The representative fluorescence images of agar phantoms with 0.166–1 mg extract/mL are presented in Figure 4A. Based on the results, it is evident that the fluorescence detection was concentration- dependent. The agar phantoms demonstrated the highest computed fluorescence profiles for 1.0 mg/mL samples, whereas the 0.166 mg/mL concentration exhibited the lowest (Figure 4A,B).

### 3.5. Bay Leaf Extracts Exhibit Tissue NIR Imaging

**Figure 5:**
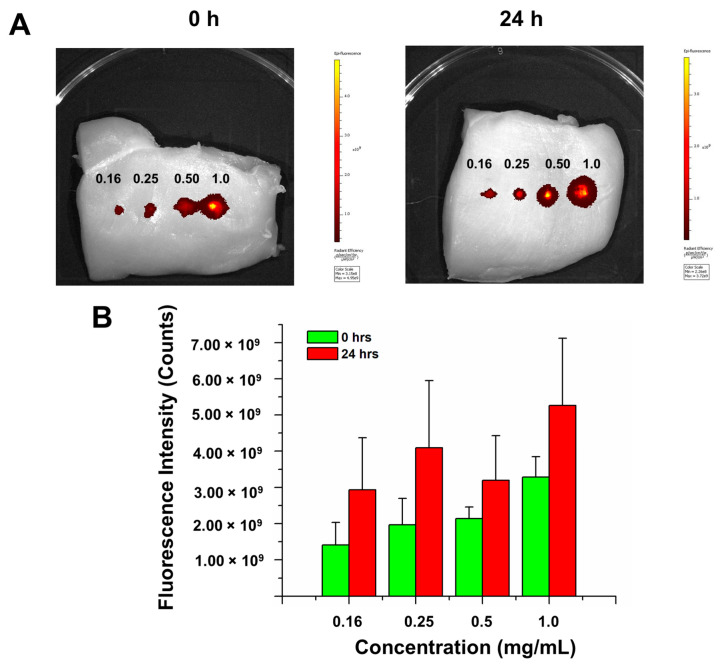
Ex Vivo tissue imaging capability of bay leaf extracts. (**A**) Physical evidence of NIR fluorescence of bay leaf extracts on chicken breast tissue at 0 time and after 24 h, and (**B**) fluorescence quantification of bay leaf extracts. Data represent the average of three different experiments.

To evaluate the tissue bioimaging capability of bay leaf extracts, an ex vivo imaging method was utilized. The bay leaf extracts of different concentrations (0.166–1.0 mg/mL) were placed on chicken tissue and fluorescence images were acquired. It is evident from Figure 5 that the bay leaf extracts exhibited a dose-dependent fluorescence imaging capability. This behavior was continued even at 24 h. These data demonstrate that bay leaf extract could act as an excellent imaging agent for improved contrast purposes in non-invasive tissue imaging applications.

### 3.6. Bay Leaf Extract Facilitates Visualization of Cancer Cells

**Figure 6:**
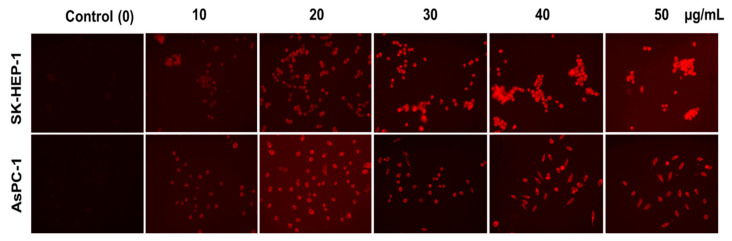
Cellular uptake of bay leaf extract in liver and pancreatic cancer cell lines. SK-HEP-1 (liver cancer) and AsPC-1 (pancreatic cancer) cell lines were used for uptake study. Cells were treated with different concentrations of bay leaf extract (contains chlorophyll) for 1 h and imaged using a fluorescent microscope. SK-HEP-1 cells showed higher uptake of chlorophyll. Images were taken at 100×.

The uptake of fluorescence probe is necessary for enhancing imaging capabilities. To examine the uptake of the bay leaf extract in cancer cells, two different cancer cell lines, SK-HEP-1 (liver cancer) and AsPC-1 (pancreatic cancer), were used. Both cell lines were exposed to different concentrations of bay leaf extract (10–50 µg/mL) for an hour and imaged with a fluorescent microscope. The results from this experiment demonstrated a concentration-dependent uptake of bay leaf extract in both cell lines; however, more significant uptake was observed in SK-HEP-1 cells than in AsPC-1 cells (Figure 6).

### 3.7. Characterization of Bay Leaf Extracts

**Figure 7:**
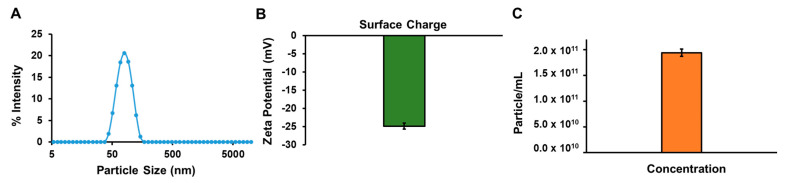
Characterization of bay leaf chlorophyll extract. Dynamic light scattering (DLS) was used to characterize Chl extracts. (**A**) Average particle size for bay leaf extracted Chl was 62.72 nm. X-axis scale was set as log. (**B**) Surface charge was recorded as −24.76 mV. (**C**) Particle concentration per mL was 1.95 × 10^11^. Data represent the average of three different experiments.

As-prepared bay leaf extracts were subjected to physical characterization; the dynamic light scattering system data exhibited the optimal average particle size of ~62.72 nm with a surface charge of ~−24.76 mV. The particle concentration was found to be 1.11 × 10^12^ particles/mL (Figure 7A–C). It was observed that this size range was smaller than other leaf extracts but slightly higher than mint extract (Table 1). The smaller particle size and negative zeta potential suggest that the bay leaf extract is stable and biocompatible as higher positive zeta potential is also known to be toxic/cytotoxic [16]. However, all selected Chl extracts had similar characteristics to bay leaf extract (Table 1). 

## 4. Discussion

The key for successful cancer management lies in its early diagnosis; hence, adequate cancer imaging is essential for improved disease control and patient survival. Biomedical imaging can be classified into optical imaging (fluorescence), magnetic resonance (MRI), and ionizing radiation (CT, PET, SPECT) based on the electromagnetic spectrum [3,17]. However, these currently used techniques have limited specificity and low sensitivity toward cancer detection, and procedures are often associated with high costs. NIR fluorescence imaging is a newer and potent modality for early cancer detection and surgery guidance. NIR imaging is of interest because of the reduced auto-fluorescent background in the biological environment and lower levels of NIR light scattering over visible light. Due to the high resolution, high sensitivity and safety, and real-time display, detailed molecular/cellular profiling can be achieved both in vitro and in vivo [18,19]. To date, there are several NIR fluorophores that have been identified, but ICG is the only NIR fluorescence contrast agent/dye for clinical use that is approved by the Food and Drug Administration. However, ICG has many drawbacks related to its poor photo- and hydrolytic stability, concentration-dependent aggregation, short half-life, and non-specific binding to proteins and tissues [20,21]. Thus, developing a safer, more photostable and biocompatible, and cancer-cell-specific NIR imaging modality is an unmet clinical need for improved early-stage cancer diagnosis. 

In this context, we utilize a natural and biocompatible NIR fluorescent probe, chlorophyll, extracted from various dietary sources. Natural chlorophyll (Chl) is an essential biomolecule for photosynthesis in plants and its molecular structure is similar to the NIR fluorescent porphyrin; therefore, Chl has various imaging and therapeutic benefits. Hence, Chl may be an ideal candidate for cellular imaging, including cancer imaging and therapy, for its non-toxicity and abundance in nature [22].

The present study shows that chlorophyll found in various dietary leaves produces superior NIR fluorescent imaging capabilities (Figure 1, Figure 2, Figure 3, Figure 4, Figure 5 and Figure 6). We further evaluated these leaf extracts through physicochemical characterization, which revealed that the extracts showed a smaller particle size and suitable negative surface charge, indicating their suitability for tissue and cellular imaging (Figure 7 and Table 1); however, based on NIR fluorescent intensity and characterization, bay leaf was selected as the best natural and dietary NIR fluorescent probe for further experiments. Additionally, our agarose phantom gel imaging data confirmed that bay leaf extracted Chl has the potential to penetrate deep inside the dense tumor tissue, as mimicked by 2.5% thick agarose gels (Figure 4) and chicken breast tissue (Figure 5). Moreover, Chl dye from bay leaf superiorly internalized in different types of cancer cells in a concentration-dependent manner without showing any aggregation or precipitation (Figure 6). Overall, data from this study promise a natural, safer, cellular NIR fluorescent imaging probe; however, additional studies are further warranted to establish this technology as a potent cellular/tissue imaging tool.

We anticipate that bay leaf extract may become a suitable medical imaging probe for the imaging of tumors in the future. As such, these extracts may not have cancer cell targeting. In this investigation, our internalization/uptake test demonstrated the ability to produce fluorescence imaging signals in the two cancer cell lines. In fact, the bay leaf extraction is also in the size range of ~62.72 nm. Recent investigations in nanobiotechnology introduced a new era of implementing natural leaf extracts for usage in biological systems towards biomedical and pharmaceutical applications. Their ultra-small particle size and their inherent physicochemical and optical properties enable their use in medical applications. The bay leaf extract produced in this study exhibits a smaller size, which is ideal for various drug delivery, nanomedicine, and theranostic applications, due to its smaller size, which can take advantage of tumor targeting via the EPR effect. However, specific tumor cell targeting could be explored through appropriate surface modification of the fluorescent ingredients of these bay leaf extracts. Our future studies will specifically focus on the uptake mechanisms and bioavailability, biodistribution, and tumor-targeted imaging capabilities of our novel, natural, near-infrared fluorescent probes. In addition, we will also develop leaf extract-based targeted nanoformulations with NIR imaging characteristics that can facilitate enhanced visualization or can illuminate tumors during surgery, offering image-guided tumor resection.

## 5. Conclusions

This study reports the feasibility of natural extracts from dietary leaves as a NIR fluorescence imaging probe. Both whole leaves and their extracts exhibit NIR fluorescence characteristics. The naturally occurring chlorophyll content in leaves is responsible for achieving excellent NIR fluorescence. Among all, bay leaf and its extract demonstrated higher fluorescence properties. The successful imaging characteristics in agarose phantom and cellular visualization of bay leaf extracts suggest its prospects in the field of fluorescence imaging. Additionally, the extracts demonstrated a unique smaller size, which can take advantage of tumor targeting via the EPR effect. Taken together, our data suggest that Chl extracted from bay leaf is a promising biocompatible alternative for NIR fluorescence that can be applied to cancer cells and tissues/tumors for enhanced detection resolution. In future work, we hope that this natural probe will be implemented as a NIR fluorescent imaging agent with an appropriate ligand or antibody conjugation for improved and tumor-targeted imaging applications.

## Figures and Tables

**Table 1:** Physicochemical characterization of six most fluorescent leaves. Particle size, zeta potential, and concentration were measured using dynamic light scattering (DLS) system. Table represents the average of three individual batches.

Samples	Size (nm)	Charge (mV)	Concentration (Particles/mL)
Bay leaf	62.72 ± 5.97	−24.76 ± 1.52	1.95 × 10^11^
Collard	170.70 ± 9.88	−21.68 ± 1.47	4.60 × 10^10^
Lettuce	131.07 ± 1.82	−25.04 ± 1.25	5.01 × 10^19^
Mint	51.8 ± 0.45	−20.87 ± 0.61	2.13 × 10^11^
Oregano	227.07 ± 2.5	−25.36 ± 0.73	9.26 × 10^9^
Spinach	75.61 ± 3.95	−21.93 ± 0.14	5.29 × 10^10^

## Supplementary Materials

The following are available online at https://www.mdpi.com/article/10.3390/jimaging7120256/s1, Figure S1: Twelve different dietary whole leaves were imaged using the IVIS Imaging System for chlorophyll content.
