# Supplementary material for: Bay Leaf Extract-Based Near-Infrared Fluorescent Probe for Tissue and Cellular Imaging
Source: J Imaging. 2021 Nov 30;7(12):256. doi: 10.3390/jimaging7120256 (PMC8705868; doi:10.3390/jimaging7120256)
Supplement: Supplementary file 1 [file jimaging-07-00256-s001.zip › jimaging-1459865-supplementary.pdf]

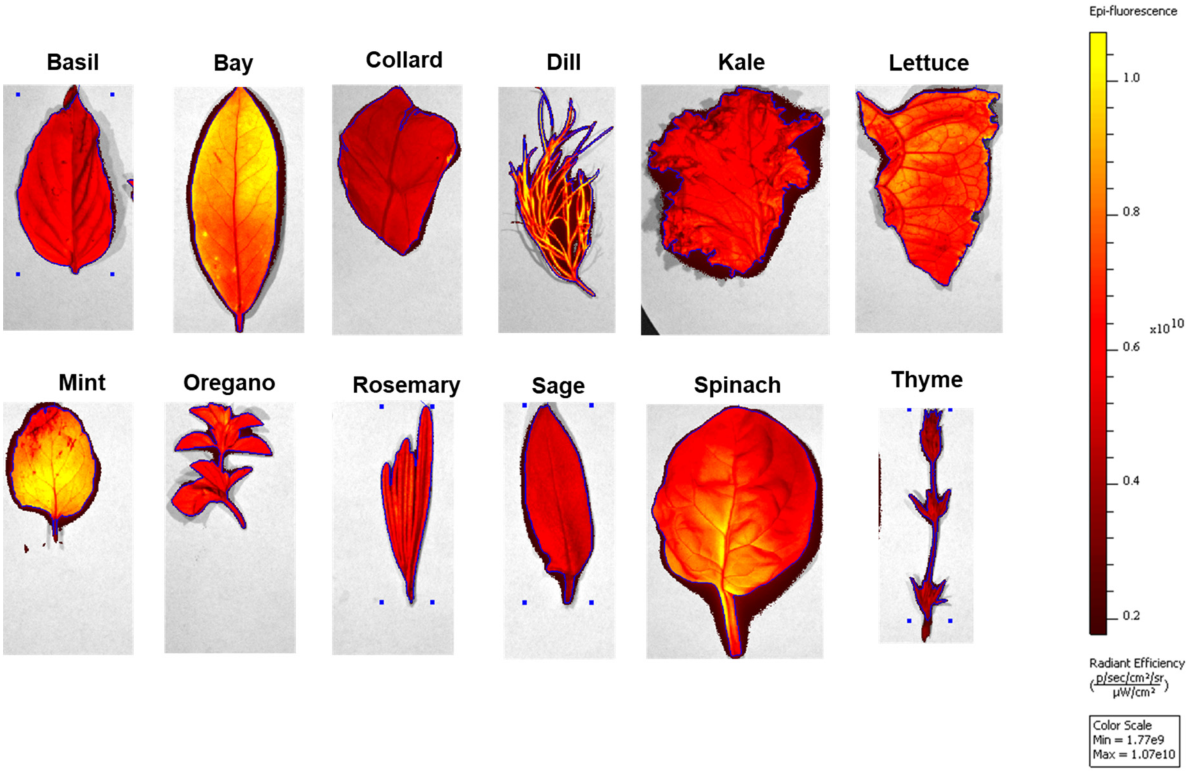

Figure S1. Twelve different dietary whole leaves were imaged using the IVIS Imaging System for chlorophyll content.
